# Analysis of Killer Immunoglobulin-Like Receptor Genes in Colorectal Cancer

**DOI:** 10.3390/cells9020514

**Published:** 2020-02-24

**Authors:** Roberto Diaz-Peña, Patricia Mondelo-Macía, Antonio José Molina de la Torre, Rebeca Sanz-Pamplona, Víctor Moreno, Vicente Martín

**Affiliations:** 1 Liquid Biopsy Analysis Unit, Oncomet, Health Research Institute of Santiago (IDIS), 15706 Santiago de Compostela, Spain; patricia_mondelo@hotmail.com; 2 Faculty of Health Sciences, Universidad Autónoma de Chile, Talca 3460000, Chile; 3 Instituto de Biomedicina (IBIOMED), CIBERESP, 24071 León, Spain; ajmolt@unileon.es (A.J.M.d.l.T.); vicente.martin@unileon.es (V.M.); 4 Group of Research on Gene-Environment-Health Interactions (GIIGAS), Universidad de León, 24071 León, Spain; 5 Unit of Biomarkers and Susceptibility, Oncology Data Analytics Program (ODAP), Catalan Institute of Oncology (ICO), Oncobell Program, Bellvitge Biomedical Research Institute (IDIBELL) and CIBERESP, L’Hospitalet de Llobregat, 08908 Barcelona, Spainv.moreno@iconcologia.net (V.M.); 6 Department of Clinical Sciences, Faculty of Medicine and Health Sciences, University of Barcelona, 08036 Barcelona, Spain

**Keywords:** KIR, colorectal carcinoma, HLA, KIR2DS3, SNP, imputation

## Abstract

Natural killer cells (NK cells) play a major role in the immune response to cancer. An important element of NK target recognition is the binding of human leucocyte antigen (HLA) class I molecules by killer immunoglobulin-like receptors (KIRs). Colorectal carcinoma (CRC) is one of the most common types of inflammation-based cancer. The purpose of the present study was to investigate the presence of KIR genes and HLA class I and II alleles in 1074 CRC patients and 1272 controls. We imputed data from single-nucleotide polymorphism (SNP) Illumina OncoArray to identify associations at HLA (HLA–A, B, C, DPB1, DQA1, DQB1, and DRB1) and KIRs (HIBAG and KIR*IMP, respectively). For association analysis, we used PLINK (v1.9), the PyHLA software, and R version 3.4.0. Only three SNP markers showed suggestive associations (*p* < 10^−3^; rs16896742, rs28367832, and rs9277952). The frequency of KIR2DS3 was significantly increased in the CRC patients compared to healthy controls (*p* < 0.005). Our results suggest that the implication of NK cells in CRC may not act through allele combinations in KIR and HLA genes. Much larger studies in ethnically homogeneous populations are needed to rule out the possible role of allelic combinations in KIR and HLA genes in CRC risk.

## 1. Introduction

Colorectal carcinoma (CRC) is a leading cancer by incidence and mortality, responsible for approximately 900,000 deaths per year worldwide [1]. CRC has long been appreciated to have a strong heritable basis, being a multifactorial entity since their pathogenesis involves both multiple genetic factors and diverse environmental factors. Large genome-wide analysis studies (GWAS) have increased our knowledge of the genetic risk factors of CRC [2,3], though much of the heritable risk of CRC remains unexplained and many rare and common variants have not yet been identified. 

**Figure 1:**
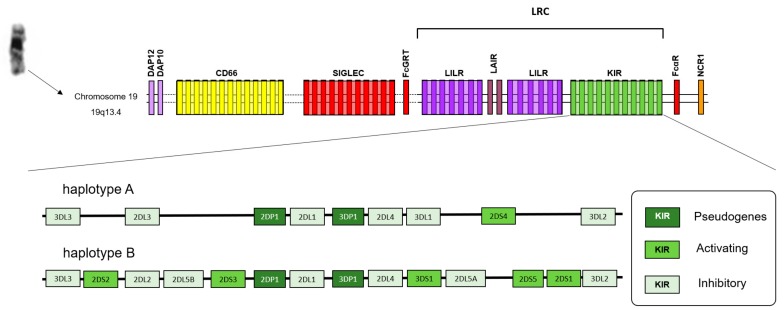
Schematic representations of the Leukocyte Receptor Complex (LRC) region and typical KIR A and B genotypes. Map of the Leukocyte Receptor Complex. The Leukocyte Receptor Complex (LRC) is formed by a cluster of genes that encode a family of proteins that contain immunoglobulin-like domains. These include the families “killer immunoglobulin-like receptors (KIR), “leukocyte immunoglobulin-like receptor” (LILR), and “leukocyte-associated immunoglobulin-like receptor” (LAIR). The “signaling lectins” (SIGLECs) and members of the family CD66 are found close to LCR.

**Figure 2:**
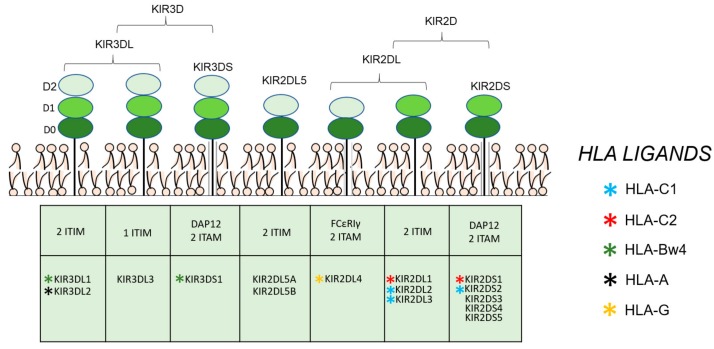
KIR protein structures and ligands. Individual KIR ligands are shown in small asterisks of different colors (KIR ligands for KIR3DL3, KIR2DL5, KIR2DS3, and KIR2DS5 are unknown).

Natural killer cells (NK cells) play a major role in the immune response to cancer [4]. An important element of NK target recognition is the binding of human leucocyte antigen (HLA) class I molecules by killer immunoglobulin-like receptors (KIRs). The KIR gene cluster is located on human chromosome 19, and constitutes a multigene family with genomic diversity with regard to gene content and allelic polymorphism [5] (Figure 1). KIRs have two (2D) or three Ig-like domains (3D), possessing a short (S) or long (L) cytoplasmic tail (Figure 2). They have been classified into two types: activating (KIR2DS and KIR3DS), where the cytoplasmic tail has the capacity to interact with activating adaptor proteins such as DAP12, delivering activating signals through their immunoreceptor tyrosine-based activating (ITAM) motif [6], and inhibitory (KIR2DL and KIR3DL) KIRs, which have one or two immunoreceptor tyrosine-based inhibition (ITIM) motifs in their cytoplasmic tail [7]. KIR2DL4 is an exception, and some reports indicate that their presence enhances NK activity and induces interferon-γ secretion [8]. Additionally, the letter P denotes pseudogenes (KIR2DP1 and KIR3DP1). Based on the genes present in each person, two basic groups of haplotypes have been proposed (Figure 1): haplotype A, that contains only one activating KIR gene, KIR2DS4; and haplotype B, with various combinations of activating KIR genes (KIR2DS1, 2DS2, 2DS3, 2DS5, 3DS1, and 2DS4), exhibiting high levels of diversity, both in terms of gene content and allelic polymorphism.

KIRs bind to recognize normally expressed HLA class I molecules (Figure 2), controlling the activation of NK cells. HLA-C alleles can be divided into two allotypes defined serologically by the Ser77/Asn80 (C1) and Asn77/Lys80 (C2) amino acid positions. Some of KIR receptors bind to HLA-C molecules [9], KIR2DL2, KIR2DL3, and KIR2DS2 interact with HLA C1, whereas KIR2DL1 and KIR2DS1 recognize HLA-C2. KIR3DL1 binds the HLA-B α1 helix around residues 76–80, with specificity for all Bw4 alleles containing isoleucine at heavy chain residue 80 [10]. Because of the relevant homology of activating KIR3DS1 to inhibitory KIR3DL1, it has been speculated that they might share the same ligand, although this assumption has not yet been determined. In vitro studies have proposed that HLA-A3 and HLA-A11 are ligands of KIR3DL2, but these interactions appear to be weak, and peptide dependent [11].

Over the last years, KIR genes have been reported strongly associated with disease susceptibility, following a model in which KIRs synergize with HLAs generating genotypes that provide different levels of activation or inhibition [5]. Recognition of HLA by KIR modulates NK function, promoting the attack to cancer cells. Therefore, variation in KIR and HLA have been thought to affect in the risk of developing cancer [12] Expression of HLA class I molecules is downregulated in more than 70% of colorectal tumors [13], and the prognostic significance of this downregulation has been reported in a large cohort of CRC cases [14]. Thus, NK cells activation by the decreased expression of HLA class I molecules on tumor cells is a relevant issue. Moreover, previous studies have focused on the connection between CRC with the polymorphism of KIR genes, some of them analyzing HLA class I presence (mainly HLA-B and HLA-C). However, these investigations are limited by small populations in different ethnic groups, providing contradictory results. In the present study, we analyzed the presence of KIR genes and HLA class I and II alleles in CRC patients and healthy controls in a larger population that is ethnically homogeneous. To the best of our knowledge, this is the largest study of KIR genes and HLA ligands in CRC to date.

## 2. Materials and Methods

### 2.1. Patients and Samples

A total of 1074 patients with CRC and 1272 healthy controls were included in the work, combining data of two case-control studies from two different locations. The first one, performed in University Hospital of Bellvitge, L’Hospitalet, Barcelona, recruited a total of 693 CRC cases and 849 healthy controls. The second study was conducted in Hospital of Leon, Leon, recruiting a total of 381 CRC cases and 423 healthy controls. Subjects were interviewed in person to gather information on risk factors and biological samples for DNA extraction were collected. Peripheral blood (27 mL) was drawn from participants, which were aliquoted in whole blood for DNA extraction, and stored at −80 °C. Saliva was collected for subjects refusing to donate blood with the Oragene® DNA Kit and stored at room temperature until DNA extraction. Standardized basic clinical and pathological information on the diagnosis of tumors was collected from hospital records by using a predefined format. The protocol was approved by the Ethics committees of the participating institutions (registration number PR149/08 for the Bellvitge University Hospital; registration number ETICA-ULE-022/2017 for University of Leon). All participants were informed about the study objectives and signed an informed consent form. Confidentiality of data was secured by removing personal identifiers in the datasets. 

### 2.2. Genotyping and Imputation

Genotyping was performed using the custom-designed 533,631 SNP Illumina OncoArray. This array was designed for cancer studies by the OncoArray Consortium, including fine-mapping of common cancer susceptibility loci with special emphasis on the HLA region, among others. Oncoarray genotyping and genotype quality controls procedure were done in the context of a large CRC genome-wide study using the OncoArray platform [2].

The classic HLA alleles at HLA–A, B, C, DPB1, DQA1, DQB1, and DRB1 were imputed using HLA Genotype Imputation with Attribute Bagging (HIBAG) and its corresponding European Ancestry reference panel [15]. SNP genotypes were phased using SHAPEIT2 [16], and the resulting phased haplotypes were uploaded to the KIR*IMP imputation server (http://imp.mcri.edu.au) [17], for KIR genes imputation.

### 2.3. Statistical Analysis

SNPs that met the quality criteria of a minor allele: a frequency of >0.01, missingness of <0.1, and/or a Hardy-Weinberg equilibrium (*p* > 0.001) were considered for inclusion in the association analyses. A total of 4874 SNPs was located on chromosome 6 (chr6: 28400339-33496193, hg19) (genotyping rate: 0.99761), whereas 791 SNPs were situated on chromosome 19 (chr19: 54500612-55599594, hg19) (genotyping rate: 0.997693). After quality control, 943 CRC cases and 1076 healthy controls were available for the association study. For single-variant association analysis (HLA and LRC regions, within chromosomes 6 and 19, respectively), we used PLINK (v1.9) [18] to perform logistic regression for binary phenotype (CRC and healthy controls). HLA association analysis was performed with the PyHLA software [19], using additive logistic regression models. The associations among KIR genes and KIR/HLA combinations with CRC were evaluated using an odds ratios (OR) estimated by logistic regression using R version 3.4.0. (https://cran.r-project.org/). For comparisons of the KIR genes frequencies, the Bonferroni correction was performed by multiplying the P value by the number of KIR genes tested (*n* = 18) to give the corrected P value (Pbonf). Age, sex, and the first two principal components of a PCA based on genetic data, were used as covariates in all tests.

## 3. Results

Table 1 displays characteristics for the CRC patients that were used for the analysis. The mean age was 69, and 64.2% of the patients were men. Most of the CRC patients (505) were current or former smoker, whereas 413 had never smoked. The CRC group did not differ from the control group with regard to any of the sociodemographic or clinical parameters included in the study (data not shown). The Tumor-Node-Metastasis (TNM) stage at diagnosis was I-II in 418 CRC patients, III in 342, and IV in 131. The location of the tumor was mainly in the rectum in 311 CRC patients (33%), sigma in 168 (17.8%), and cecum in 98 (10.4%).

We considered genotypic associations in the HLA and LRC regions based on SNP genotyping. Only three markers showed suggestive associations (*p* < 10^−3^), all of them located in intergenic regions: rs16896742 (*p* = 3.97 × 10^−4^), rs28367832 (*p* = 6.57 × 10^−5^), and rs9277952 (*p* = 4.01 × 10^−5^) (Table 2, Supplementary Files 1 and 2). Then, we proceeded to analyze associations based on HLA imputation. Table 3 shows the frequency distribution between CRC cases and controls of HLA alleles (top ten associations sorted by p-value). In the unadjusted regression model, some HLA alleles showed a significant association with CRC. Specifically, HLA-C*07:01 allele frequency was decreased in patients with CRC compared to healthy controls (*p* < 0.05, OR = 0.82, 95% CI 0.69–0.98), while HLA-C*12:03 and HLA DRB1*11:01 alleles was increased in CRC compared to controls (*p* < 0.05, OR = 1.30, 95% CI 1.01-1.67; and *p* < 0.05, OR = 1.32, 95% CI 1.06-1.65, respectively). However, when p-values were corrected and adjusted for false discovery rate (FDR) using the Benjamini-Hochberg test, no differences were observed in the distribution of the rest of HLA class I and II alleles among controls and CRC patients.

The frequency of each KIR gene in CRC patients and healthy controls is shown in Table 4. The frequency of KIR2DS3 was significantly increased in the CRC patients compared to healthy controls (39.24% vs. 32.53%, *p* < 0.005, Pbonf < 0.05, OR = 1.34, 95% CI 1.12-1.61). No other KIR gene showed different frequencies between cases with CRC compared with controls. Since KIR2DL3 and KIR2DL2 are considered alleles, as well as KIR3DL1 and KIR3DS1, we also tested for each gene pair the distribution of the homozygous and heterozygous genotypes of both alleles [20] (Table 5). Next, we analyzed the different interactions of KIR genes with and without their HLA ligands (Table 5). None of these analyses showed significant differences between any group of CRC patients and controls.

## 4. Discussion

Different studies have shown conflicting results of specific KIR genes with HLA alleles and cancer susceptibility [12]. Regarding KIR/HLA and CRC, several genetic associations have been described, but not widely replicated (Table 6). Already in 2007, Middleton et al. reported no association of KIR with CRC in Europeans [21]. Analyzing the distribution of HLA-C1 and C2 groups, they found a significant difference in the CRC group. However, any theory was speculative because of the small number of samples taken into account. They only included 81 CRC patients and 100 healthy controls. Also, Al Omar et al. described a lack of association of KIR and CRC [22], although they showed a strong association of the presence of HLA-Bw4 in Caucasian population (128 CRC patients and 255 healthy controls). A study performed in Koreans, in 241 CRC patients and 159 healthy controls, showed that the frequency of KIR2DS5 was significantly increased in CRC patients [23]. They also reported that the frequencies of KIR3DL1, KIR2DS2 and KIR2DS4 were decreased in CRC patients. Moreover, KIR2DS2/HLA-C1 combination presented a protective effect in CRC susceptibility. In a preliminary study performed in the Saudi population [24] (52 CRC patients and 70 controls), was reported an increase of activating KIR2DS1, KIR2DS5 and KIR3DS1 in patients with CRC. A meta-analysis of four independent studies including a total of 470 individuals with CRC and 483 individuals in control group [25], indicated that CRC was affected by KIR2DS5. After, a report of 2016 performed in Brazilian CRC Caucasoid population [26], 154 CRC cases and 216 controls, showed no significant differences for HLA ligands and KIR genes between groups, but the Bx haplotypes (AB and BB) were more frequent in controls compared to in CRC patients. Recently, another study with an Iranian population examined a total of 165 patients with CRC as well as 165 healthy controls [27], showed that possessing more inhibitory KIR genes was a potential risk to CRC while genotypes with many activating KIR genes was associated with protection against it. Despite all bibliography, the role of KIR variability in cancer, CRC included, remains unclear, mainly due to the smaller number of studies involving large and well-characterized cohorts.

In light of such discrepancies and considering the need for larger sample size studies, this study was conducted with a large and ethnically homogeneous population for CRC patients and healthy controls. Only KIR2DS3 exhibited suggestive association, showing that their frequency was significantly increased in patients with CRC compared with control subjects. The associations of the remaining KIR genes included in the study were not significant. In a previous study published by Beksac et al. [28] KIR2DS3 showed a similar distribution between CRC cases and control (39.1% vs. 33.8%, respectively), which was not significant because of the sample size. Moreover, the study showed that KIR2DS3 was associated with protection from recurrence. We could not assess this, since we have not yet performed a follow-up of the CRC patients to know their evolution. KIR2DS3 is an NK cell associated gene, that functions, for example, as host risk factor predicting failure to spontaneously clear Hepatitis C virus (HCV) virus by immune mediated mechanisms [29]. Also, KIR2DS3 has been associated with fatal outcome in Ebola virus infection [30]. However, there is currently no known function for KIR2DS3, and evidence suggests that it is not expressed at the cell surface [31]. We think that KIR2DS3 could be a genetic marker for a closely related linked gene that could be responsible of the biological effect. Obviously, further research is required to confirm this assertion.

We tested the effects of KIR genes in combination with their natural ligand, specifically: KIR3DL1 and HLA-Bw4I80 (also KIR3DS1); and combinations of KIR2DL1, 2DL2, 2DL3, and 2DS1, with HLA-C groups (C1 and C2). However, despite a large number of comparisons performed, none of these tests showed significant changes between CRC and healthy controls. The activation profile of KIRs is genetically determined in each individual and leads to diverse levels of functionality in NK cells. Thus, many works describe associations of combinations KIR/HLA and disease, proposing that compound genotypes provide different levels of activation or inhibition for NK and T cells. We rule out this possibility in CRC, at least in Caucasoid populations.

The HLA region plays a crucial role in numerous pathologies, as it accounts for 25% of known associations from the GWAS catalog (https://www.ebi.ac.uk/gwas/), especially with immune-related diseases. However, the link between HLA and CRC is unknown. There are data showing that the expression of HLA class II molecules on CRC cells is involved in its pathogenesis [32]. Specifically, presence of particular DRB1 alleles influence the susceptibility to ulcerative colitis-associated CRC [33]. Aureli et al. described that the DRB1*13:01 and DRB1*11:01 alleles were associated with an increased and reduced risk to develop CRC [34], respectively. Although the number of CRC patients analyzed is limited for this study (*n* = 53), it may provide a starting point. However, we observed an inverse relationship between the allele frequency of DRB1*11:01 and CRC, increased in cases compared to controls. Moreover, this association was not confirmed when p-value was corrected. We cannot exclude the association, as DRB1*11:01 allele has been associated with a variety of malignancies, including breast cancer and hairy cell leukaemia [35,36], but at least their implication is not due to the participation of any KIR receptor.

Several limitations in our design should be kept in mind. The imputation of HLA alleles may be controversial due to the complexity of the HLA region. Although SNP association studies importantly expanded in the last decade, direct HLA allele association has been hindered by the complexity of typing. HLA imputation offers a statistical alternative to current HLA typing, cutting costs, and time alike. HLA imputation will be important in the context of association studies [37]. Moreover, although most current results are negative, we believe that our data may add significantly to the available literature in the field. We described the possible implication of KIR2DS3 in the susceptibility to CRC, at least involving a genetic marker linked to a potential causal variant. We also showed that the implication of NK cells in CRC could not be related to specific associations between KIR/HLA combinations. It may be a mistake to believe that single KIR receptor/HLA ligand can lead or influence the function of NK cells in this regard. In summary, we show the need to conduct studies in larger and ethnically homogeneous populations in order to reach robust conclusions.

## Figures and Tables

**Table 1:** Baseline characteristics of the patient population.

	CRC Patients (*n* = 943)	Healthy Controls (*n* = 1076)
Age (median, range)	69 (23–91)	64.15 (24–92)
**Gender** Female Male	338 (35.8%) 605 (64.2%)	527 (48.98%) 549 (51.02%)
**Smoke**		
Current smoker Former smoker Never smoker Unknown	119 (12.6%) 386 (40.9%) 413 (43.8%) 26 (2.8%)	180 (16.73%) 335 (31.13%) 553 (51.39%) 8 (0.74%)
**TNM stage at diagnosis**		
I–II III IV Unknown	418 (44.3%) 342 (36.3%) 131 (13.9%) 52 (5.5%)	
**Tumor location**		
Rectum Sigma Cecum Ascending colon Sigmoid colon Descending colon Others* Unknown	311 (33%) 168 (17.8%) 98 (10.4%) 81 (8.6%) 70 (7.4%) 52 (5.5%) 152 (16.1%) 11 (1.2%)	

*colon unspecified, recto-sigma, rectosigmoid junction, sigmoid colon, hepatic flexure, splenic flexure and transvers colon.

**Table 2:** SNP association values (only *p* values < 10^−3^ are shown) based on the allele frequencies characterized for the CRC patients vs. healthy controls.

Chr	SNP	Position	Associated Gene	A1	MAF CRC Cases	MAF Healthy Controls	*p* Value	OR
6	rs16896742	29922740	Intergenic	G	0.31	0.3669	3.97 × 10^−4^	0.79
6	rs28367832	31305731	Intergenic	A	0.42	0.4845	6.57 × 10^−5^	0.77
6	rs9277952	33204274	Intergenic	A	0.15	0.1097	4.01 × 10^−5^	1.47

A1: minor allele nucleotide; CRC: colorectal carcinoma; MAF: minor allele frequency; SNP: single nucleotide polymorphism; OR: odds ratio.

**Table 3:** Frequency distribution of HLA alleles between CRC cases and controls.

HLA Allele	AF CRC Cases	AF Healthy Controls	*P* value	OR	95% CI	Padj
DRB1*11:01	0.1002	0.0785	0.0137	1.32	1.06–1.65	0.0961
C*07:01	0.1304	0.1543	0.0321	0.82	0.69–0.98	0.1774
C*12:03	0.0689	0.0534	0.0443	1.30	1.01–1.67	0.1774
A*02:01	0.2264	0.2519	0.0576	0.87	0.75–1.00	0.3453
A*29:02	0.0848	0.0730	0.1554	1.18	0.94–1.50	0.4662
DRB1*04:01	0.0467	0.0562	0.1751	0.82	0.62–1.09	0.6128
B*35:01	0.0583	0.0511	0.3096	1.15	0.88–1.52	0.8689
C*16:01	0.0843	0.0757	0.3136	1.13	0.89–1.42	0.6060
B*44:03	0.1071	0.0976	0.3240	1.11	0.90–1.35	0.8689
C*07:02	0.0817	0.0897	0.3641	0.90	0.72–1.13	0.6060

AF: allele frequency; OR: odds ratio; CI: confidence interval; Padj: p-values adjusted and corrected for FDR using the Benjamini-Hochberg test.

**Table 4:** KIR gene and genotype frequencies in CRC patients and healthy controls.

	Healthy Controls		CRC Cases				
*KIR* Gene	n	%	n	%	*p*-value *	Pbonf	OR (95% CI)
KIR2DL1	1075	99.91	942	99.89	NS	NS	
KIR2DL2	490	45.54	418	44.33	NS	NS	
KIR2DL3	925	85.97	818	86.74	NS	NS	
KIR2DL4	1076	100	943	100	NS	NS	
KIR2DL5	500	46.47	450	47.72	NS	NS	
KIR3DL1ex4	1030	95.72	911	96.61	NS	NS	
KIR3DL1ex9	1032	95.91	911	96.61	NS	NS	
KIR3DL2	1076	100	943	100	NS	NS	
KIR2DS1	386	35.87	346	36.69	NS	NS	
KIR2DS2	530	49.26	480	50.9	NS	NS	
KIR2DS3	350	32.53	370	39.24	0.002	0.036	1.34 (1.12–1.61)
KIR2DS4total	1030	95.72	909	96.39	NS	NS	
KIR2DS4wt	366	34.01	306	32.45	NS	NS	
KIR2DS4del	905	84.11	801	84.94	NS	NS	
KIR2DS5	286	26.58	257	27.25	NS	NS	
KIR3DS1	337	31.32	319	33.83	NS	NS	
KIR2DP1	1074	99.81	943	100	NS	NS	
KIR3DP1	1076	100	943	100	NS	NS	
**KIR Genotype**	**n**	**%**	**n**	**%**	*p*-value *		**OR (95% CI)**
AA	280	26.02	243	26.77	NS		
Bx	796	73.98	700	74.23	NS		

NS: not significant; OR: odds ratio; CI: confidence interval. Pbonf: *P* value using Bonferroni correction. * Two-tailed Fisher’s exact test.

**Table 5:** Distribution of HLA ligands frequencies and KIR-HLA interactions in CRC cases and healthy controls.

	Healthy Controls		CRC Cases			
HLA Ligands	N	%	n	%	*p*-Value	OR (95% CI)
Bw4	726	67.47	650	68.93	NS	
Bw6	885	82.25	766	81.23	NS	
Bw4/Bw4	191	17.75	177	18.77	NS	
Bw4/Bw6	535	49.72	473	50.16	NS	
Bw6/Bw6	350	32.53	293	31.07	NS	
Bw4I80	416	38.66	361	38.28	NS	
Bw4T80	402	37.36	373	39.55	NS	
HLA-C1	890	82.71	752	79.75	NS	
HLA-C2	725	67.38	640	67.87	NS	
HLA-C1C1	351	32.62	303	32.13	NS	
HLA-C1C2	539	50.09	449	47.61	NS	
HLA-C2C2	186	17.29	191	20.25	NS	
**KIR genotypes**	**N**	**%**	**n**	**%**	***p*-value**	**OR (95% CI)**
KIR3DL1/KIR3DL1	738	68.59	624	66.17	NS	
KIR3DL1/KIR3DS1	294	27.32	287	30.43	NS	
KIR3DS1/KIR3DS1	43	4.00	32	3.39	NS	
KIR2DL2/KIR2DL2	149	13.85	125	13.26	NS	
KIR2DL2/KIR2DL3	341	31.69	293	31.07	NS	
KIR2DL3/KIR2DL3	584	54.28	525	55.67	NS	
					NS	
**KIR ligand associations**	**N**	**%**	**n**	**%**	***p*-value**	**OR (95% CI)**
KIR3DS1-Bw4I80	131	12.17	114	12.09	NS	
KIR3DS1-Bw4T80	125	11.62	123	13.04	NS	
KIR3DS1-Bw4	228	21.19	206	21.85	NS	
KIR3DS1-Bw6	285	26.49	264	27.99	NS	
KIR3DL1-Bw4I80	402	37.36	349	37.01	NS	
KIR3DL1-Bw4T80	386	35.87	356	37.75	NS	
KIR3DL1-Bw4	697	64.78	626	66.38	NS	
KIR3DL1-Bw6	847	78.72	742	78.69	NS	
KIR2DL1/HLA-C1C1	351	32.62	302	32.03	NS	
KIR2DL1/HLA-C1C2	538	50.00	449	47.61	NS	
KIR2DL1/HLA-C2C2	186	17.29	191	20.25	NS	
KIR2DL2/HLA-C1C1	149	13.85	125	13.26	NS	
KIR2DL2/HLA-C1C2	189	17.57	153	16.22	NS	
KIR2DL2/HLA-C2C2	152	14.13	140	14.85	NS	
KIR2DL3/HLA-C1C1	339	31.51	297	31.50	NS	
KIR2DL3/HLA-C1C2	403	37.45	337	35.74	NS	
KIR2DL3/HLA-C2C2	183	17.01	184	19.51	NS	
KIR2DS1/HLA-C1C1	115	10.69	102	10.82	NS	
KIR2DS1/HLA-C1C2	194	18.03	175	18.56	NS	
KIR2DS1/HLA-C2C2	77	7.16	69	7.32	NS	

NS: not significant; OR: odds ratio; CI: confidence interval.

**Table 6:** Basic research studies showing the associations between KIR and HLA in CRC patients.

Reference	Type of Experiment/Objective	Conclusions
[19]	109 CRC patients (70 bladder and 34 laryngeal) and 100 controls. HLA and KIR genotyping.	No differences in KIR/HLA frequencies was observed between patients and controls.
[20]	128 CRC patients and 255 controls. KIR and HLA genotyping.	The data showed no significant differences between KIR gene frequencies in CRC patients versus controls.
[21]	241 CRC patients and 159 controls from Korean populations. KIR and HLA-C genotyping	The activating KIR2DS5 was more frequent in Korean CRC patients, showing a risk for the disease. The frequencies of KIR3DL1, KIR2DS2 and KIR2DS4 were lower in the rectal cancer subgroup, and they could have a protective effect against CRC. Also, the lower frequency of KIR2DS2 in patients with HLA-C1 homozygote, may be a protective effect too.
[22]	52 CRC patients and 70 controls from Saudi population. KIR and HLA-C genotyping.	Activating KIRs (2DS1, 2DS2, 2DS3, 2DS5 and 3DS1) was more frequent in CRC patients, suggesting their presence a risk for disease.
[23]	470 CRC patients and 483 controls. KIR genotyping.	The presence of KIR2DS5 was associated with CRC like as a non-protective gene. This result explains the inflammatory basis of this cancer.
[24]	154 CRC patients and 216 controls from Caucasian Brazilian population. KIR and HLA genotyping.	No associations between KIRs and HLA in CRC was observed. However, the Bx haplotype was more frequent in controls than in patients, being a possible mechanism of protection to CRC.
[25]	165 colorectal adenocarcinoma patients and 165 controls. KIR genotyping.	The presence of activating KIRs (≥ 4) and KIR3DL1, 3DS1, 2DS1 and 2DS4, were associated with protection against metastasis in CRC patients.
[26]	29 CRC recurrent patients (in 5 years) vs. 58 CRC non-recurrent patients (in 5 years) after surgery and 154 controls. KIR and HLA-class I genotyping.	The increment of activating KIRs (in particularly 2DS2 and 2DS3) and the lack of inhibitory KIRs (in particularly 2DL1) was associated with long term disease-free survival and this was independent of tumor localization or stage. Also, HLA-A-Bw4 was associated with recurrent disease.

## Supplementary Materials

The following are available online at https://www.mdpi.com/2073-4409/9/2/514/s1, Supplementary File 1: SNP association values based on the allele frequencies characterized for the CRC patients vs. healthy controls (HLA region); Supplementary File 2: SNP association values based on the allele frequencies characterized for the CRC patients vs. healthy controls (LRC region).
